# Development and Validation of the Adolescent Media Health Literacy Scales: Rasch Measurement Model Approach

**DOI:** 10.2196/35067

**Published:** 2022-04-15

**Authors:** Sasha A Fleary

**Affiliations:** 1 Department of Community Health and Social Sciences CUNY Graduate School of Public Health and Health Policy New York, NY United States

**Keywords:** adolescents, health communications, health literacy, measurement, media health literacy, Rasch, mobile phone

## Abstract

**Background:**

High media use has been implicated in negative social and health outcomes among adolescents. Therefore, it is critical that adolescents develop skills to healthily engage with media content. Media health literacy (MHL), skills for assessing and responding to health-related media content, and potentially targetable moderators for the relationship between media use and health-related outcomes are understudied in adolescents. The lack of MHL assessment tools may have contributed to this research gap.

**Objective:**

This study aimed to develop and validate test-based scales of adolescents’ MHL.

**Methods:**

The items developed were vetted iteratively via community reviews and cognitive interviews to establish content and face validity. Adolescents (N=355) completed a questionnaire that included the revised MHL items. The scales (Recognition/Identification, Influence/Critical Analysis, and Action/Reaction) were validated using Rasch measurement models. Convergent validity was assessed by correlating the summed scores of the three scales with existing functional and internet-related health literacy measures. Criterion validity was assessed by modeling logistic regressions for predicting health literacy–related behaviors from each scale after controlling for demographics. Effect sizes were estimated, and a short form was also validated.

**Results:**

The final MHL scales (Recognition/Identification, Influence/Critical Analysis, and Action/Reaction) fit their Rasch models. The 9-item Recognition/Identification and 9-item Influence/Critical Analysis scales had good convergent validity with functional and internet-related health literacy measures and were positively related to reading instructions before taking medicines and questioning the truthfulness of health information found online. The 12-item MHL Scales-Short Form also had good convergent and criterion validity. However, convergent and criterion validity were not established for the 3-item Action/Reaction Scale.

**Conclusions:**

The Recognition/Identification and Influence/Critical Analysis scales and the MHL Scales-Short Form may be used to determine the impact of MHL on media use and health outcome relationships and ultimately inform the development of interventions and policies to affect these relationships in multiple settings.

## Introduction

### Background

The presence of digital media is evolving as people, especially adolescents, continue to socialize and interact with the world more frequently through this medium [1]. Twenge et al [2] found that the time 12th graders spent online more than doubled from 2006 to 2016, and 82% of 12th graders used social media daily in 2016. According to a Pew survey (2018), approximately 95% of adolescents own or have access to a smartphone, and almost half of them are online constantly [3]. High levels of media use among adolescents are related to negative outcomes, including poor academic achievement [4], obesogenic behaviors and obesity [5], mental health problems [6], and substance use [7]. Media literacy, and media health literacy (MHL) specifically, may mitigate these negative relationships. Few studies have explored the effect of media literacy on health beliefs and health outcomes [8-10], and even fewer studies have examined the effect of MHL specifically on these outcomes [11].

Media literacy is the ability to access, understand, evaluate, scrutinize, and create print and electronic media [12,13]. MHL differs from media literacy in that it is more specific to how one engages with health-related media content. Levin-Zamir et al [11] proposed a conceptualization of MHL that was influenced by the functional (reading and writing skills required for everyday situations), communicative or interactive (skills to draw meaning from multiple types of communication and apply to situations), and critical (critical analysis of information and skills to foster sociopolitical action) health literacy (HL) domains proposed by Nutbeam [14,15]. Levin-Zamir et al [11] described MHL as including the following four domains: the identification and recognition of health-related media content, the assessment of health-related media content’s intended influence on behavior, the critical analysis of health-related media content, and the declaration of intent to act in response to health-related media content.

The paucity of health behavior research on MHL in comparison with the amount of such research on media literacy is likely due to the lack of measures for assessing adolescents’ MHL. Levin-Zamir et al [11] developed a measure of MHL that includes the four domains described in their definition of the concept. However, the items were based on video segments that adolescents viewed, including qualitative and quantitative responses, and the sample was restricted to Jewish adolescents in Israel. Therefore, the measure would be difficult to use in most research and clinical settings, and its applicability and utility outside of a Jewish Israeli population is unclear. There are some measures of media literacy that are specific to health behaviors that are not MHL. For example, Primack et al [16] developed a measure to assess adolescents’ smoking content–related media literacy. However, more general measures of adolescents’ MHL are necessary to assess these important skills across multiple health behaviors.

### This Study

Guided by the definition and measure of MHL provided by Levin-Zamir et al [11], this study aims to develop and validate test-based scales of MHL that could be administered and scored in research and clinical settings. This study used the Rasch measurement model, a probabilistic model that tests data fit against a measurement model rather than a sampled population, as is characteristic of classical test theory [17]. Thus, the resulting fit statistics and validated scales are not sample dependent [18]. In the Rasch measurement model, the probability of a specific person responding in a specific manner to a specific item is calculated, and persons with higher abilities have higher probabilities of endorsing higher items, whereas items with higher difficulties have a lower probability of being endorsed [17,18]. Item difficulty and personal ability are estimated independent of the sample and items in the scale, respectively [18]. This methodology is appropriate for validating the MHL scales, as it identifies the person abilities level and cutoff scores distinguish between different levels of ability that are informative when trying to assess and intervene on skills. We hypothesized that the final scales would have good convergent validity with previously validated measures of functional and internet-related HL and would also demonstrate good criterion validity with self-reported HL-related behaviors.

## Methods

### Study Design

A multiphase mixed methods design was used to develop and validate the Adolescent MHL Scales.

### Ethics Approval

This study was approved by the Tufts University Social Behavioral and Educational Institutional Review Board (approval number: 1411003). Informed consent was obtained from college students. Parent permission and adolescent assent were obtained for adolescents’ participation in data collection.

### Measures

#### Demographics

Participants self-reported their age, gender (male, female, transgender, nonbinary, and other), ethnicity (Hispanic, Latino or Latina, or Spanish origin), and race (Black or African American, Asian, Native American or Alaskan Native, Native Hawaiian or other Pacific Islander, White, and other). Given the small sample size, Asian, Native American or Alaskan Native, and Native Hawaiian or other Pacific Islander were combined. Participants who selected multiple races were labeled as multiracial. All questions included a “prefer not to answer” option.

#### Newest Vital Sign

The Newest Vital Sign (NVS [19]) is a commonly used measure of functional HL and has good internal consistency (Cronbach *α*=.76). The NVS includes 6 reading and numeracy questions related to a provided nutritional facts label. Responses were scored, summed, and categorized as a high likelihood of limited literacy (0-1 correct responses), a possibility of limited literacy (2-3 correct responses), and adequate literacy (≥4 correct responses). Summed scores were used to evaluate convergent validity between the functional HL and MHL scales.

#### eHealth Literacy Scale

The eHealth Literacy Scale (eHEALS [20]) is a measure of internet-related HL with good internal consistency (Cronbach *α*=.88). The 8-item measure assesses individuals’ comfort with, knowledge of, and perceived skills for accessing, evaluating, and using health information found on the internet. Response options were scored on a 5-point Likert scale ranging from strongly disagree to strongly agree. Summed scores were used to evaluate convergent validity between the internet-related HL and MHL scales.

### HL Behaviors

Items that were examples of adolescents’ applied use of their HL skills were developed for this study. These items were informed by focus groups where adolescents described how they used their HL skills [21]. Participants indicated whether they engaged in 2 behaviors indicative of HL—questioning the truthfulness of health information found online and reading instructions before taking medicines. These items were consistent with the scope and reach of the applied use aspect of HL conceptualized by Sørenson et al [22].

### MHL Scales Development

Measurement development involved item bank development, quantitative data collection, and measurement evaluation.

#### Item Bank Development

**Figure 1:**
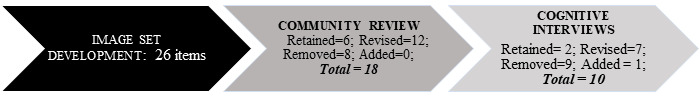
Illustration of iterative image bank revisions before large scale quantitative data collection.

Using the definition and measure of MHL provided by Levin-Zamir et al [11] as a guide, 26 images were created to assess participants’ ability to recognize health messages in media, of which 10 (47%) were intentionally unrelated to health. We chose to use images rather than videos because images are ubiquitous across multiple media outlets, including the social media platforms that adolescents frequent (eg, Instagram), health websites and clinics (via infographics), and in the community (eg, health information posters at school and print advertisements). Images (vs videos) were also chosen because they allowed for self-administration and quick scoring. The 26 images were piloted with undergraduate research assistants who were not involved in this project as a community review step, given this demographic’s use of media is similar to that of adolescents. Their feedback was used to revise 12 (46%) images and remove 8 (31%) images. The 18-image measure (including 6 images unrelated to health) was then piloted. In all, 19 cognitive interviews were conducted with college students (age: mean 18.74, SD 0.99 years; women: n=14, 73%; Black participants: n=2, 10%; Asian participants: n=4, 21%) to gather feedback on the appropriateness and relatability of the images, to gather suggestions for modifications, and to qualitatively assess participants’ MHL according to the four domains—recognition/identification, influence, critical analysis, and action/reaction—proposed by Levin-Zamir et al [11]. Data collection from the cognitive interviews concluded when saturation was achieved. The qualitative responses were transcribed and content-analyzed. The images were modified based on the content analysis. Specifically, approximately 7 (39%) images were revised (text was removed and images were modified), 3 (17%) health-related images and all 6 (33%) non–health-related images were removed, and 1 (6%) image was added (Figure 1). Non–health-related images were removed, as responses varied in cognitive interviews based on how participants defined health. Qualitative responses were also used to create response options for questions related to influence, critical analysis, and action. It should be noted that only images with consistent responses across interviewees were chosen for these additional questions for the measure. The revised measure contained 10 health-related images. Each image included an accompanying question about health-related message recognition, and 3 (30%) images included 14 questions on influence, critical analysis, and action/reaction.

#### Quantitative Data Collection and Measurement Evaluation

The revised measure was administered to a convenience sample of adolescents (aged 12-18 years), and Rasch measurement models were used to identify the items that best fit the latent constructs. In coordination with the head health teacher at a local high school, adolescents were recruited via flyers that were posted in school common areas and provided to them, as well as classroom announcements, and they completed the survey during their health class. Data from students whose parents signed permission forms and who signed assent forms were retained and used in this study (N=355). The survey was administered electronically on researcher-provided tablets using the Qualtrics survey platform (Qualtrics International Inc). Students received a US $15 gift card for their participation.

### Statistical Analyses

Rasch models were estimated in Winsteps (version 5.1.1) [23], and all other analyses were conducted in SPSS (version 27; IBM Corporation) [24]. The full measure (24 items) was first analyzed using the Rasch Partial Credit Model, as response options were dichotomous and polytomous. The Rasch Partial Credit Model allows each item to have its own rating scale structure; therefore, not all items have to be on the same rating scale. As anticipated, analyzing the measure as a single latent construct revealed multidimensionality. Separate clustering was observed on the standardized residual contrast plot for action/reaction-oriented items and recognition-oriented items, and the remaining items were clustered together. Given the consistency with the clusters with the a priori content writing of the items (informed by Levin-Zamir et al [11]), the clusters were evaluated as separate scales. Recognition/identification items were analyzed using the Rasch Dichotomous Model, and influence/critical analysis and action/reaction items were analyzed using the Rasch Partial Credit Model, as they included polytomous responses.

The key assumptions of Rasch include unidimensionality (“Do items assess a shared latent construct?”), local independence (“Are the item responses statistically independent of each other?”), and monotonicity of the latent trait (“Are scores monotonically nondecreasing across the latent trait?”). Unidimensionality was evaluated by examining the principal component analysis of the residuals [25] and was confirmed if the eigenvalue of the unexplained variance in the first contrast was <2 [26]. On the basis of the recommendation by Christensen et al [27], the Q_3,*_ test statistic, which is calculated as *Q_3,max_* (maximum standardized residual correlation between a pair of items) minus the mean of *Q_3,_* (mean of all standardized residual correlations between item pairs), was calculated. *Q_3,max_* and the Q_3,*_ test statistic were compared with the critical values reported by Christensen et al [27] to determine if there was local independence. Critical values for *Q_3,max_* and the Q_3,*_ test statistic at the 99th percentile were 0.24 and 0.31, respectively. Monotonically ascending test characteristic curves were indicative of monotonicity [28].

Person and item parameters were estimated using joint maximum likelihood estimation procedures. Outfit mean squares for person and item parameters were examined for good fit (0.5-1.5=good fit; <0.5 or 1.6-2.0=unproductive but not degrading to the measure) [29]. If items had outfit mean squares of >1.5, the standardized statistics were then examined. Items with standardized statistics of >2 were considered for removal. Items with outfit mean squares of <0.5 are less concerning; therefore, they were not considered for removal [30]. The refinement of the measures was performed iteratively. Items with the highest mean square outfit misfit and standardized outfit statistics of >2 were removed first, and the models were re-estimated and re-evaluated after each removal. Regarding person misfit, for each analysis, 1 round of the most misfitting responses was removed (taken from tables of the most misfitting responses), and the models were re-estimated and compared with the original models. If removing these responses did not improve the model fit, the original model was retained, but if the model fit improved, the model with the removed responses was retained for final analyses [29]. Negative point-measure correlations were removed, as these indicated that the items did not belong to the scale [25,31]. Similar to other studies using the Rasch measurement model, final decisions to retain or remove items were based on statistical findings and theoretical reasonings for the items [32]. The key assumptions of the Rasch models were examined at each iteration of model estimation.

Reliability for both items and persons were examined. For items, item separation reliability statistics closer to 1 indicated good item separation (ie, good item difficulty range). Rasch person reliability and classical test theory reliability statistics assume symmetric ability, which is rarely the case in health-related research. To address this, Wright [33] proposed an alternative method of calculating reliability; the Wright sample-independent reliability statistic is computed once measurement calibration is complete [33]. The calculations involve determining the number of strata across the scores and then using this to calculate the sample-independent reliability (ie, number of levels^2^/1 + number of levels^2^). Sample-independent reliability was appropriate for this study because the sample was skewed in terms of ability. Uniform differential item functioning (DIF) for gender, age, and ethnicity was also calculated to determine whether the items performed similarly across subpopulations. Detecting statistically significant DIF that is ≥0.5 logits requires at least 100 participants per subgroup [34], and significance thresholds are typically set to *P*<.01 to account for multiple tests. Given the small sample sizes, age was grouped into early (aged 12-15 years) and late (aged 16-18 years) adolescence to calculate DIF. Sample size requirements were met for all analyses; Rasch model calculations can be estimated with 99% confidence within 0.5 logits with a minimum sample size of 108 to 243 [35], and each response category surpassed the minimum requirement of 10 responses for polytomous items [36].

Descriptive statistics were calculated after the three scales were finalized. Convergent validity (whether 2 measures of constructs that should be related are related [37]) was assessed by correlating the summed scores of the three scales with existing functional and internet-related HL measures. The correlations were expected to be significant but in the low to moderate range, given that functional and internet-related HL are related but have different constructs from those of MHL (ie, hetero-trait). Criterion validity (whether the score on 1 measure is related to a direct outcome of the phenomenon [38]) was assessed by modeling logistic regressions for predicting HL-related behaviors from each scale after controlling for demographics. Effect sizes were also estimated by estimating receiver operating characteristic curves and transforming the areas under the curves to Cohen *d* values by using the tables proposed by Salgado [39].

### Unplanned Post Hoc Analyses

Although the initial intent of the measure development process was to develop scales to assess the MHL domains outlined by Levin-Zamir et al [11], the resultant two scales with good validity would likely be difficult to administer in most settings because of the length of the scales. Furthermore, having a single score for MHL may be more useful and easier to interpret in some settings. Therefore, an additional Rasch model was estimated only for images for which all questions were asked (images MHLH6, MHLH7, and MHLH8) in an attempt to create a short form. Items were only included in the short-form estimation if they were included in the final versions of the two validated scales. All of the above outlined procedures were followed to determine the validity of the short form.

## Results

### Overview

A sample of 355 adolescents (age: mean 16, SD 1.34 years; adolescent girls: n=165, 46.5%) completed the survey. All but 1 participant chose either the male or female option. Approximately 147 (41.8%) adolescents in the sample were non-Hispanic or non-Latinx, and the largest racial group was other (approximately 27.3%), partially owing to Hispanic and Latinx adolescents choosing “other” as their race. A subsample (n=200) of adolescents completed the NVS; 70 (35%) of these adolescents had a high likelihood of limited literacy, and 54 (27%) had adequate literacy (see Table 1 for additional descriptive statistics).

**Table 1:** Charactertistics of the sample (N=355).

Variable			Values, n (%)		Recognition/ Identification						Influence/ Critical Analysis							Action/ Reaction							Short Form			
					Values, mean (SD)		*F* value			Values, mean (SD)			*F* value			Values, mean (SD)				*F* value			Values, mean (SD)				*F* value	
**Gender**								2.21						12.65^a^							1.04							18.31^a^
	Boys	136 (38.3)		6.81 (1.98)					11.47 (2.55)						3.55 (2.53)							13.83 (2.83)						
	Girls	165 (46.5)		7.12 (1.48)					12.49 (2.23)						3.86 (2.57)							15.18 (2.36)						
	Missing	54 (15.2)		—^b^					—						—							—						
**Age (years)**								0.24						0.31							1.90							0.40
	12-14	57 (16.1)		6.89 (1.78)					12.08 (2.75)						4.43 (2.41)							14.63 (3.01)						
	15	50 (14.1)		6.98 (1.49)					11.91 (2.38)						3.79 (2.15)							14.49 (2.59)						
	16	63 (17.7)		7.02 (1.89)					12.24 (2.33)						3.43 (2.79)							14.81 (2.53)						
	17	101 (28.5)		7.09 (1.80)					11.91 (2.35)						3.35 (2.59)							14.42 (2.66)						
	18	34 (9.6)		6.77 (1.54)					11.70 (2.78)						4.03 (2.61)							14.10 (2.79)						
	Missing	50 (16.1)		—					—						—							—						
**Hispanic or Latinx**								0.30						1.26							0.02							0.47
	Yes	150 (42.3)		6.98 (1.67)					11.81 (2.44)						3.68 (2.57)							14.40 (2.68)						
	No	147 (41.4)		7.09 (1.67)					12.14 (2.50)						3.72 (2.52)							14.62 (2.73)						
	Missing	58 (16.3)		—					—						—							—						
**Race**								1.71						2.10^c^							1.23							2.56^d^
	ANAANNHOPI^e^	24 (6.8)		6.38 (2.32)					10.86 (3.30)						2.79 (2.34)							13.14 (3.52)						
	Black	61 (17.2)		6.87 (1.68)					11.91 (2.76)						3.74 (2.57)							14.27 (2.96)						
	White	66 (18.6)		7.37 (1.56)					12.34 (2.10)						3.79 (2.73)							15.03 (2.37)						
	Multiracial	31 (8.7)		6.97 (1.71)					12.67 (2.25)						3.37 (2.28)							15.03 (2.53)						
	Other^f^	97 (27.3)		7.08 (1.54)					12.00 (2.14)						4.01 (2.58)							14.69 (2.32)						
	Missing	76 (21.4)		—					—						—							—						
**Newest Vital Sign**								11.13^a^						31.93^a^							0.65							29.33^a^
	High likelihood of limited literacy	70 (19.7)		6.47 (1.98)					10.24 (2.60)						3.94 (2.33)							12.68 (3.03)						
	Possibility of limited literacy	76 (21.4)		6.96 (1.53)					12.28 (2.12)						3.81 (2.75)							14.75 (2.24)						
	Adequate literacy	54 (15.2)		7.85 (0.92)					13.35 (1.57)						3.41 (2.74)							16.06 (1.63)						
	Missing	155 (43.7)		—					—						—							—						
**Recognition/Identification**								330.19^a^						27.63^a^							1.83							53.72^a^
	Emerging	26 (7.3)		3.00 (1.23)					9.43 (2.66)						2.96 (2.18)							10.76 (3.00)						
	Expanding	257 (72.4)		7.38 (1.17)					12.23 (2.32)						3.71 (2.60)							14.87 (2.42)						
	Missing	72 (20.3)		—					—						—							—						
**Influence/Critical Analysis**								9.66^a^						336.96^a^							3.45^d^							283.96^a^
	Emerging	20 (5.6)		5.82 (2.65)					6.10 (1.25)						3.25 (2.36)							8.00 (1.71)						
	Expanding	184 (51.8)		6.96 (1.61)					11.42 (1.64)						3.46 (2.43)							13.93 (1.81)						
	Bridging	89 (25.1)		7.55 (1.14)					14.37 (0.49)						4.28 (2.77)							17.06 (0.74)						
	Missing	62 (17.5)		—					—						—							—						
**Action/Reaction**								3.31^c^						8.76^g^							670.44^a^							10.16^g^
	Emerging	193 (54.4)		6.91 (1.70)					11.65 (2.62)						2.11 (1.43)							14.15 (2.87)						
	Expanding	106 (29.9)		7.29 (1.59)					12.54 (2.11)						6.52 (1.35)							15.20 (2.19)						
	Missing	56 (15.8)		—					—						—							—						
**Short Form**								17.15^a^						219.50^a^							1.35							277.97^a^
	Emerging	6 (1.7)		4.67 (2.58)					4.67 (1.21)						2.00 (1.67)							6.00 (1.10)						
	Expanding	157 (44.2)		6.75 (1.76)					10.72 (1.95)						3.75 (2.45)							13.08 (1.99)						
	Bridging	122 (34.4)		7.58 (1.12)					13.95 (0.75)						3.72 (2.74)							16.75 (0.77)						
	Missing	70 (19.7)		—					—						—							—						

^a^*P*<.001.

^b^Not available (missing data).

^c^*P*<.10.

^d^*P*<.05.

^e^ANAANNHOPI: Asian, Native American/Alaskan Native, Native Hawaiian/other Pacific Islander.

^f^In all, 70 adolescents who identified as other indicated that they were Hispanic/Latinx.

^g^*P*<.01.

### Recognition/Identification

The Recognition/Identification item bank contained 10 items, and 9 (90%) items were retained for the final scale (Multimedia Appendix 1). The final scale assessed adolescents’ ability to identify health-related messages in images. One item was removed because of high outfit statistics. The removal of the most misfitting person responses improved the model and item fit; therefore, the final model was estimated after removing these misfitting responses. Point-measure correlations for the final scales were between 0.45 and 0.61, suggesting high correlations with person abilities. The assumptions of unidimensionality (eigenvalue=1.5), local independence (*Q_3,max_*=0.17; Q_3,*_ test statistic=0.27), and monotonicity were met. No DIF was detected for gender, age, or ethnicity. Item separation reliability (0.98) was acceptable. The Wright sample-independent reliability statistic was 0.80, and the scores differentiated 2 distinct levels of performances—emerging (scores of 0-4) and expanding (scores of 5-9). The Kuder-Richardson Formula 20 (KR-20) *α* was .74 (see Table 2 for the fit statistics).

**Table 2:** Rasch item difficulties and fit statistics ordered from the most to least difficult item on each individual scale.

Item			Media health literacy individual scales								
			Difficulty		SE		Outfit MNSQ^a^		Outfit ZSTD^b^		PMC^c^
**Recognition/Identification**											
	MHLH1REC	3.09		0.16		1.16		0.85		0.58	
	MHLH10REC	1.53		0.15		0.92		−0.68		0.61	
	MHLH8REC	1.47		0.15		1.22		1.88		0.51	
	MHLH9REC	0.34		0.17		0.87		−0.78		0.57	
	MHLH3REC	−0.04		0.18		1.01		0.10		0.52	
	MHLH4REC	−0.49		0.20		0.72		−1.18		0.54	
	MHLH2REC	−1.84		0.30		0.39		−1.46		0.51	
	MHLH7REC	−2.02		0.32		0.55		−0.79		0.45	
	MHLH6REC	−2.05		0.32		0.28		−1.68		0.51	
**Influence/Critical Analysis**											
	MHLH7AGR	1.42		0.07		0.90		−1.08		0.68	
	MHLH6AGR	1.05		0.07		0.98		−0.22		0.73	
	MHLH7INT	0.79		0.15		1.15		1.28		0.39	
	MHLH6CON	0.12		0.16		1.20		1.15		0.36	
	MHLH8INF	−0.39		0.20		1.08		0.39		0.29	
	MHLH7CON	−0.51		0.20		0.72		−1.16		0.65	
	MHLH7INF	−0.57		0.20		1.16		0.68		0.26	
	MHLH6INT	−0.86		0.22		0.64		−1.26		0.33	
	MHLH6INF	−1.05		0.24		0.55		−1.49		0.35	
**Action/Reaction**											
	MHLH7ACT	0.21		0.09		0.86		−1.72		0.79	
	MHLH8ACT	0.09		0.09		1.12		1.33		0.79	
	MHLH6ACT	−0.30		0.09		0.97		−0.31		0.81	

^a^MNSQ: mean square.

^b^ZSTD: standardized statistic.

^c^PMC: point-measure correlation.

Recognition/Identification scores (mean 6.99, SD 1.73) differed significantly by NVS category. Specifically, adolescents who had adequate literacy on the NVS had higher Recognition/Identification scores than those who had a high likelihood of limited literacy (mean difference=1.38; *P*<.001) or the possibility of limited literacy (mean difference=0.89; *P*=.006). The scale had convergent validity with the NVS (*r*=0.30; *P*<.001) and eHEALS (*r*=0.22; *P*=.001). Regarding criterion validity, the scale was positively related to adolescents questioning the truthfulness of health information found online (odds ratio [OR] 1.40, 95% CI 1.17-1.66; *P*<.001; Cohen *d*=0.47) and reading instructions before taking medicines (OR 1.34, 95% CI 1.09-1.66; *P*=.006; Cohen *d*=0.34).

### Influence/Critical Analysis

The Influence/Critical Analysis item bank contained 11 items, and 9 (89%) items were retained for the final scale (Multimedia Appendix 2). The final scale assessed adolescents’ ability to correctly identify the content and intent of the messages and their critical analyses on the intended influences of the messages. In all, 2 (11%) items were removed because of high outfit statistics. The removal of the most misfitting person responses did not improve the model fit. The point-measure correlations for the final scale were between 0.26 and 0.73. The assumptions of unidimensionality (eigenvalue=1.6), local independence (*Q_3,max_*=0.15; Q_3,*_ test statistic=0.23), and monotonicity were met. No DIF was detected for gender, age, or ethnicity. Item separation reliability (0.96) was acceptable. The Wright sample-independent reliability statistic was 0.90, and the scores differentiated 3 distinct levels of performances—emerging (scores of 0-7), expanding (scores of 8-13), and bridging (scores of 14-15). The KR-20 *α* was .91. The possible scores ranged from 0 to 15 rather than 0 to 9 because this scale included dichotomous and polytomous items and, for the Rasch Partial Credit Model, each polytomous response option has a unique score that corresponds to the degree of correctness (see Table 2 for fit statistics).

Influence/Critical Analysis scores (mean 11.95, SD 2.48) differed by gender and NVS category. Adolescent girls scored significantly higher than adolescent boys (mean difference=1.02; *P*=.001), and adolescents who had adequate literacy on the NVS had higher Influence/Critical Analysis scores than those who had a high likelihood of limited literacy (mean difference=0.60; *P*<.001) or the possibility of limited literacy (mean difference=0.25; *P*=.029). Convergent validity with the NVS (*r*=0.49; *P*<.001) and eHEALS (*r*=0.22; *P*=.001) was established. Regarding criterion validity, the scale was positively related to questioning the truthfulness of health information found online (OR 1.34, 95% CI 1.18-1.52; Cohen *d*=0.69) and reading instructions before taking medicines (OR 1.31, 95% CI 1.11-1.54; Cohen *d*=0.86).

### Action/Reaction

The Action/Reaction item bank contained 3 items that assessed adolescents’ intention to take personal or social action in reaction to health-related content in the media image. The response options were ranked from no action to public and personal action intended/planned. All items were retained for the final scale (Multimedia Appendix 3). There were no misfitting items, and the removal of the most misfitting person responses did not improve the model fit; therefore, all items and responses were retained. Point-measure correlations for the final scale were between 0.79 and 0.81. The assumptions of unidimensionality (eigenvalue=1.6), local independence (*Q_3,max_*=−0.60; Q_3,*_ test statistic=−1.10), and monotonicity were met. No DIF was detected for gender, age, or ethnicity. Item separation reliability was low (0.82). The Wright sample-independent reliability statistic was 0.80, with the scores differentiating 2 distinct levels of performances—emerging (scores of 0-4) and expanding (scores of 5-9). The KR-20 α value was .71 (see Table 2 for fit statistics).

Action/Reaction scores (mean 3.68, SD 2.53) did not differ according to demographic characteristics or the NVS category. The scale was significantly positively correlated with the NVS (*r*=0.24; *P*=.002) but not significantly correlated with eHEALS scores; therefore, convergent validity was established only for functional HL. Regarding criterion validity, Action/Reaction scores were not significantly related to measured HL-related behaviors.

### MHL Scales-Short Form

All 12 items of the MHL Scales-Short Form comprised 1 dimension, and all items fit the Rasch Partial Credit Model (Multimedia Appendix 4). Point-measure correlations were between 0.26 and 0.67. The assumptions of unidimensionality (eigenvalue=1.72), local independence (*Q_3,max_*=0.14; Q_3,*_ test statistic=0.20), and monotonicity were met. No DIF was detected for gender, age, or ethnicity. Item separation reliability (0.96) was acceptable. The Wright sample-independent reliability statistic was 0.90, with the scores differentiating 3 distinct levels of performances—emerging (scores of 0-7), expanding (scores of 8-15), and bridging (scores of 16-18). The KR-20 *α* value was .93 (see Table 3 for fit statistics).

**Table 3:** Rasch item difficulties and fit statistics ordered from most to least difficult item on the Media Health Literacy Scales-Short Form.

Item	Media Health Literacy Scales-Short Form				
	Difficulty	SE	Outfit MNSQ^a^	Outfit ZSTD^b^	PMC^c^
MHLH8REC	1.48	0.14	1.19	2.21	0.36
MHLH7AGR	1.44	0.07	1.04	0.44	0.64
MHLH6AGR	1.16	0.06	0.83	−1.52	0.67
MHLH7INT	0.80	0.15	1.09	0.73	0.39
MHLH6CON	0.16	0.16	1.08	0.53	0.35
MHLH8INF	−0.35	0.20	0.94	−0.18	0.28
MHLH7CON	−0.45	0.19	0.65	−1.58	0.64
MHLH7INF	−0.53	0.20	1.13	0.55	0.26
MHLH7REC	−0.79	0.22	0.98	0.03	0.28
MHLH6INT	−0.81	0.22	0.65	−1.25	0.31
MHLH6INF	−1.00	0.24	0.53	−1.60	0.34
MHLH6REC	−1.13	0.24	0.57	−1.32	0.32

^a^MNSQ: mean square.

^b^ZSTD: standardized statistic.

^c^PMC: point-measure correlation.

The MHL Scales-Short Form scores (mean 14.50, SD 2.70) differed by gender, race, and NVS category. Adolescent girls scored higher than adolescent boys (mean difference 1.34; *P*<.001), and White adolescents had higher scores than adolescents in the Asian, Native American or Alaskan Native, and Native Hawaiian or other Pacific Islander cluster. For the NVS categories, adolescents who had adequate literacy had higher MHL scores than those who had a high likelihood of limited literacy (mean difference=3.38; *P*<.001) or the possibility of limited literacy (mean difference=1.31; *P*=.008). Convergent validity with the NVS (*r*=0.48; *P*<.001) and eHEALS (*r*=0.21; *P*=.002) was established. Regarding criterion validity, the scale was positively related to questioning the truthfulness of health information found online (OR 1.31, 95% CI 1.16-1.47; Cohen *d*=0.68) and reading instructions before taking medicines (OR 1.32, 95% CI 1.13-1.53; Cohen *d*=0.91). Tables showing *Q_3_*_,_ matrices and reliability statistics for all scales are included in Multimedia Appendix 5.

## Discussion

### Principal Findings

This study developed and validated test-based scales of adolescents’ MHL. Face and initial content validity were established using community reviews and cognitive interviews. The final scales fit their respective Rasch models and met the assumptions of unidimensionality, local independence, and monotonicity required for Rasch models. Criterion and convergent validity were established for the Recognition/Identification and Influence/Critical Analysis scales and their combined short form (MHL Scales-Short Form). For Action/Reaction, only convergent validity with functional HL was established.

The questions on the Recognition/Identification scale tested adolescents’ ability to recognize that the image was health-related but did not address a more nuanced interpretation of the images. Conversely, the more specific questions on the Influence/Critical Analysis scale focused on the complexity of engaging with health-related media messages, namely the initial interpretation of the content (content question), followed by understanding the purpose behind the message (intent or influence) and the adolescents’ level of agreement with the message (agreement). Given that HL is developmental [22], responses to these items will be strongly influenced by adolescents’ experiences with media and health content as well as their capacity for critical thought, drawing on previous knowledge and integrating multiple sources of information. Therefore, although all responses for some items (eg, influence items) may seem plausible based on an adolescent’s background, adolescents whose responses reflect more critical thought and/or the integration of multiple sources of information would have higher influence/critical analysis skills and are more likely to choose responses that are scored higher on the scale.

Although the Action/Reaction scale was validated using the Rasch Partial Credit Model, convergent validity was only established with functional HL, and criterion validity was not established. The items in this scale are qualitatively different from the other items, as this scale attempts to assess intended personal and community advocacy in reaction to health-related media content. Our scoring system ranked individuals’ responses from no action to personal and community action. It is possible that our criterion validity items were not sufficiently sensitive or specific to detect the validity of this scale. It is also possible that the items may not adequately assess the Action/Reaction construct as intended. Furthermore, asking adolescents to predict what they may do might be too abstract, and this approach might be highly susceptible to social desirability responses based on what is the *right thing to do*. Alternative items or methods for assessing this concept (eg, more detailed scenarios for the media content) should be explored, and the expansion of the items (eg, more empowerment-related HL behaviors) should also be considered to improve the validity of this scale for measuring this construct. This scale should not be used until further refinement and evaluation of the psychometric properties are performed.

The items were originally written to align with the definition and measure of MHL provided by Levin-Zamir et al [11]. However, the measure resulted in 3 scales rather than 4. Levin-Zamir et al [11] conceptualized the domains of recognition/identification and influence as being similar to the functional HL proposed by Nutbeam [14]. They also equated their critical analysis domain (agreement with content) with the critical HL proposed by Nutbeam [14]. However, Nutbeam [14] described interactive HL as skills that can be used “to extract information and derive meaning from different forms of communication”; therefore, both the influence and critical analysis domains in the definition given by Levin-Zamir [11] are better aligned with interactive HL. Consistent with the definition of interactive HL by Nutbeam [14], the influence and critical analysis items in our item bank formed 1 dimension that may be better explained as interactive MHL, and the recognition items formed a separate dimension that may be better explained as functional MHL. Relatedly, Nutbeam [14] described the goal of critical HL as personal and community empowerment. The domain of action/reaction proposed by Levin-Zamir et al [11] focuses on the intent to engage in action as a result of the health message and equates this to the interactive HL proposed by Nutbeam [14], but the definition and question items are arguably better aligned with the critical HL proposed by Nutbeam [14].

It is possible that the use of images rather than videos, all close-ended responses rather than open- and close-ended responses, and the Rasch measurement for analyses rather than the Guttman scale may have contributed to differences in the final MHL scales when compared with the Levin-Zamir et al [11] measure. However, conceptually, the items on each scale are what would be expected if the definitions of functional, interactive, and critical HL proposed by Nutbeam [14] were applied to MHL, and the same item bank was used. Furthermore, the use of images rather than videos has practical implications for how the scales may be used. The capability for self-administration in multiple modalities (eg, online and on paper) means that the scales would have higher utility in research and practice settings. In addition, images such as those used in the MHL scales are familiar to adolescents and are present in multiple types of media that adolescents frequent for health and non–health-related content (eg, websites, health clinics, school hallways, and social media).

The effect sizes for predicting HL-related behaviors from the Recognition/Identification and Influence/Critical Analysis scales ranged from small to large (Cohen *d*=0.34-0.86), suggesting that the final scales are useful in predicting HL-related behavior and for assessing the MHL skills necessary for engaging in applied HL behaviors. The smallest effect sizes were noted for Recognition/Identification; however, this is not surprising, given that this domain is similar to functional HL and is a more basic skill set than interactive HL. If both scales cannot be used, the MHL Scales-Short Form should be used, as it includes both recognition/identification and influence/critical analysis items. Furthermore, given that the effect sizes for predicting HL-related behaviors from the short form ranged from medium to large, the short form is as good an indicator as or a better indicator of HL-related behaviors than either scale alone.

### Limitations

This study has several limitations. The sample comprised adolescents enrolled in health classes, with some having an interest in health careers; therefore, their MHL ability might be higher than the average adolescent. However, to account for the skewed sample, sample-independent reliability was used instead of person reliability statistics that assume a normal distribution of ability. Future studies with normally distributed ability levels should continue to assess the validity of the scales. Another limitation was the insufficient age and race subgroup sample sizes for calculating DIF for each age and racial group. Despite the insufficient subgroup samples for calculating DIF, Rasch analyses were conducted with an adequate sample, and the racial diversity of the participants throughout each phase of the study is a significant strength of this study. Future studies should include appropriate sample sizes to determine measurement invariance for multiple demographic variables related to MHL, including parent education, household income, and chronic disease status. Longitudinal designs are also required to assess the predictive validity and the sensitivity and specificity of the scales to detect changes over time. An important future consideration is the validation of these MHL scales or the development of similar scales for assessing MHL in adults. The infiltration and expansion of fake news and misinformation on media platforms, especially those related to health, have led to poor and misinformed health decision-making with potentially grave consequences. Although MHL has been implicated in individuals using and sharing health misinformation, there are no measures of adult MHL for assessing this implication or identifying individuals who may benefit from an MHL intervention.

### Conclusions

This study developed test-based scales of adolescents’ MHL that may be self-administered. The Rasch measurement model supported a 9-item Recognition/Identification scale, a 9-item Influence/Critical Analysis scale, a 3-item Action/Reaction scale, and a 12-item Short-Form including items from the Recognition/Identification and Influence/Critical Analysis scales. Although all scales met the assumptions of the Rasch measurement model, the Action/Reaction scale did not have good convergent and criterion validity; therefore, this scale should not be used until more research is done on its psychometric properties. The Recognition/Identification and Influence/Critical Analysis scales and the MHL Scales-Short Form had good criterion and convergent validity. These scales could be used in clinical and research settings to inform interventions, policies, and programs to improve adolescents’ MHL and health decision-making.

### Practical Implications

The development of MHL scales is a critical step in determining the impact of MHL on the relationship between media use and health outcomes and ultimately informing the development of programs, interventions, and policies to reduce the negative effect of media use on adolescents’ health outcomes. The Recognition/Identification and Influence/Critical Analysis scales and their combined short form are useful in multiple settings. For example, health teachers may use the scales as a pretest to assess their students’ abilities and to plan and implement curricula for improving students’ MHL accordingly. Researchers and practitioners may also use the scales to identify MHL intervention needs for adolescents. Furthermore, the scales may be used to collect data to establish a baseline understanding of adolescents’ MHL skills, which may inform health-related media content developed for adolescents.

## Appendix

Multimedia Appendix 1 Final Recognition/Identification Scale.

Multimedia Appendix 2 Final Influence/Critical Analysis Scale.

Multimedia Appendix 3 Final Action/Reaction Scale.

Multimedia Appendix 4 Media Health Literacy Scales-Short Form.

Multimedia Appendix 5 Tables of standardized residual correlations and reliability statistics of all media health literacy (MHL) scales.

## Abbreviations

DIF: differential item functioningeHEALS: eHealth Literacy Scale
HL: health literacy
KR-20: Kuder-Richardson Formula 20
MHL: media health literacy
NVS: Newest Vital Sign
OR: odds ratio
