# Supplementary material for: Development and Validation of the Adolescent Media Health Literacy Scales: Rasch Measurement Model Approach
Source: JMIR Pediatr Parent. 2022 Apr 15;5(2):e35067. doi: 10.2196/35067 (PMC9055475; doi:10.2196/35067)
Supplement: Multimedia Appendix 1 [file pediatrics_v5i2e35067_app1.docx]

Appendix I. Final Recognition/Identification Scale

The correct answers are highlighted.

Instructions: Look at these images and answer the questions that follow.

| **#** | **VARIABLE NAME** | **DESCRIPTION** | **ANSWER CHOICES** |
| --- | --- | --- | --- |
| **1** | **MHLH1REC** | *Use the image below to answer the next question.*  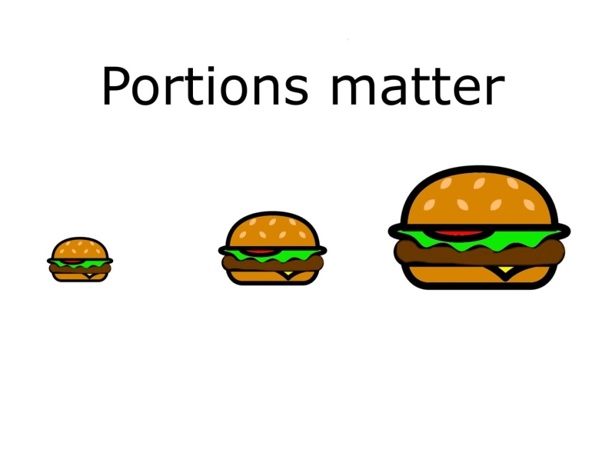  Is there a health-related message in the picture? | 1: Yes  0: No |
| **2** | **MHLH2REC** | *Use the image below to answer the next question.*  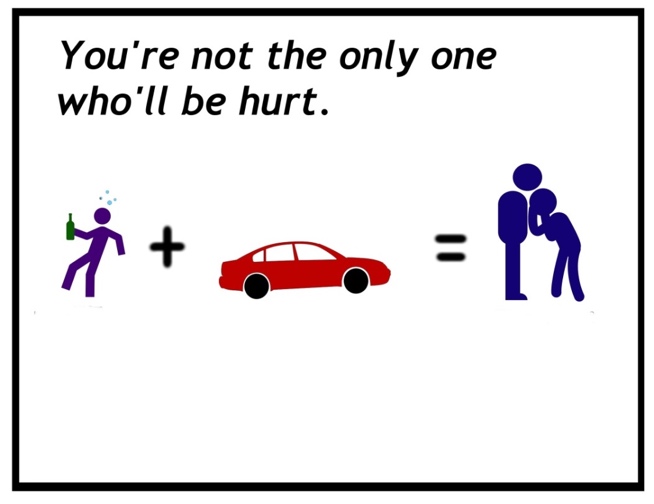  Is there a health-related message in the picture? | 1: Yes  0: No |
| **3** | **MHLH3REC** | *Use the image below to answer the next question.*  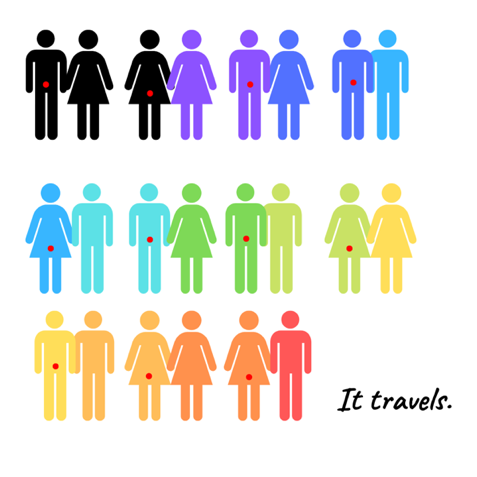  Is there a health-related message in the picture? | 1: Yes  0: No |
| **4** | **MHLH4REC** | *Use the image below to answer the next question.*  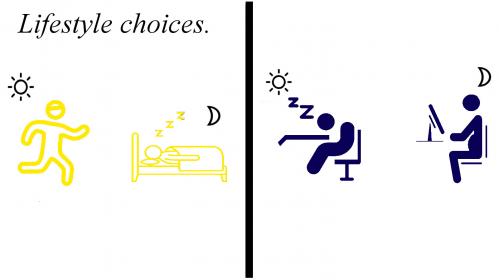  Is there a health-related message in the picture? | 1: Yes  0: No |
| **5** | **MHLH6REC** | *Use the image below to answer the next question.*  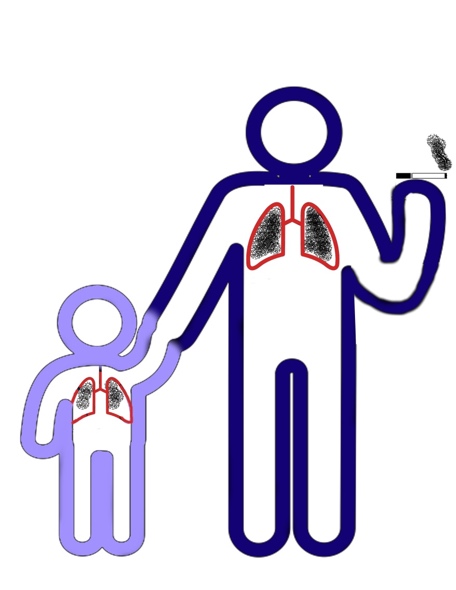  Is there a health-related message in the picture? | 1: Yes  0: No |
| **6** | **MHLH7REC** | *Use the image below to answer the next question.*  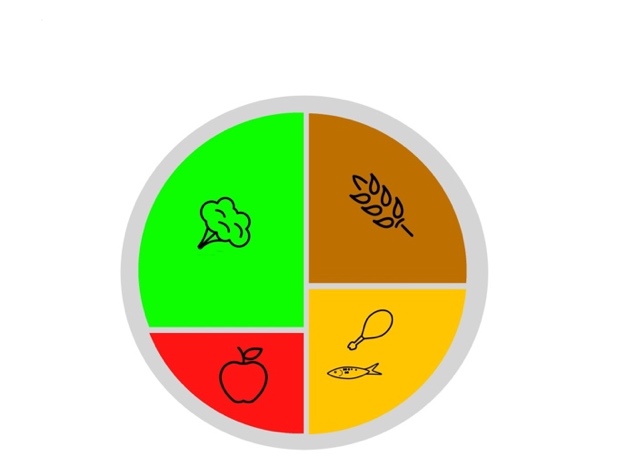  Is there a health-related message in the picture? | 1: Yes  0: No |
| **7** | **MHLH8REC** | *The following is an image of a store-front in a low-income community. Use the image to answer the next question.*  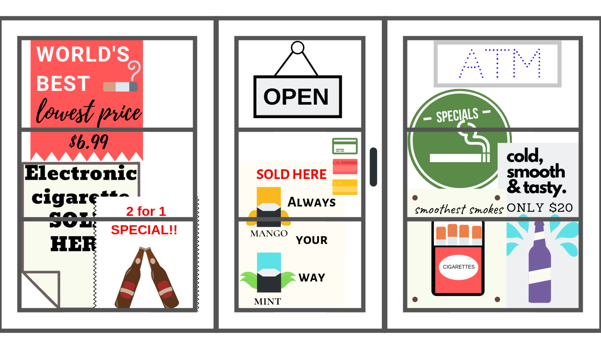  Is there a health-related message in the picture? | 1: Yes  0: No |
| **8** | **MHLH9REC** | *Use the image below to answer the next question.*  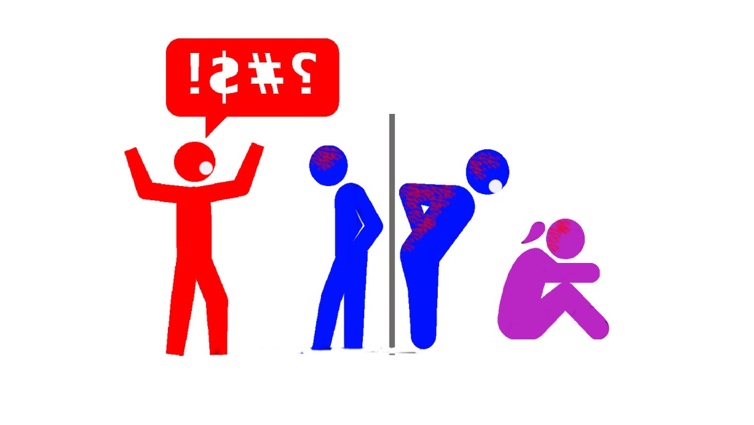  Is there a health-related message in the picture? | 1: Yes  0: No |
| **9** | **MHLH10REC** | *Use the image below to answer the next question.*  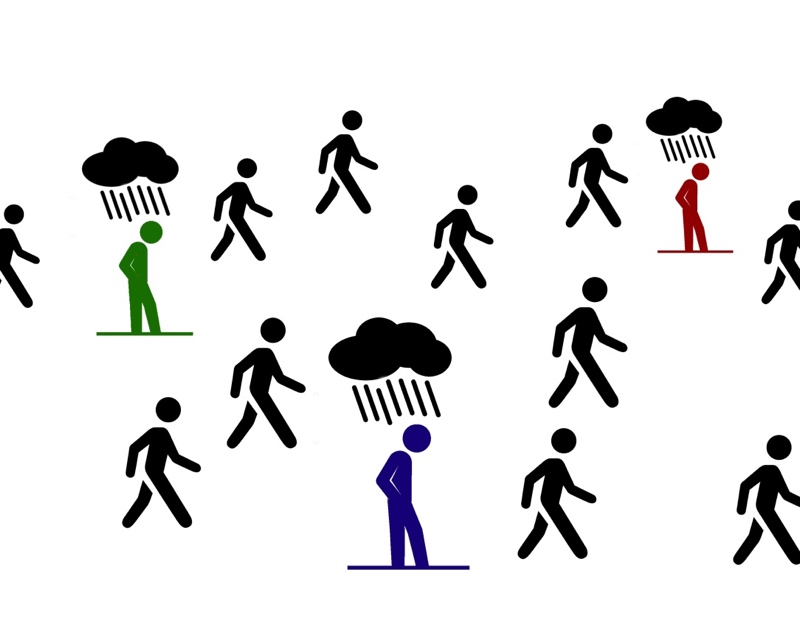  Is there a health-related message in the picture? | 1: Yes  0: No |
