# Supplementary material for: Development and Validation of the Adolescent Media Health Literacy Scales: Rasch Measurement Model Approach
Source: JMIR Pediatr Parent. 2022 Apr 15;5(2):e35067. doi: 10.2196/35067 (PMC9055475; doi:10.2196/35067)
Supplement: Multimedia Appendix 3 [file pediatrics_v5i2e35067_app3.docx]

Appendix III. Final Action/Reaction Scale

| **#** | **VARIABLE NAME** | **DESCRIPTION** | **ANSWER CHOICES** | **SCORING** |
| --- | --- | --- | --- | --- |
|  |  | *Use the image below to answer the next question.*  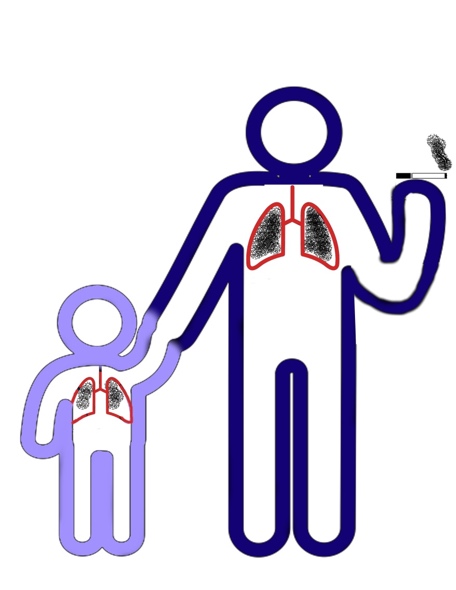 |  |  |
| **1** | **MHLH6ACT** | What is your personal reaction to the message? | 1: No action planned  2: Intention/plans to change personal behavior  3: Intention/plans to reinforce personal behavior  4: Intention/plans to take public action to change or reinforce behavior of others  5: Intention/plans to take public action and change or reinforce behavior of self and others | IF 1, Scored = 0.  IF 2 or 3, Scored = 1.  IF 4, Scored = 2.  IF 5, Scored = 3. |
|  |  | *Use the image below to answer the next question.*  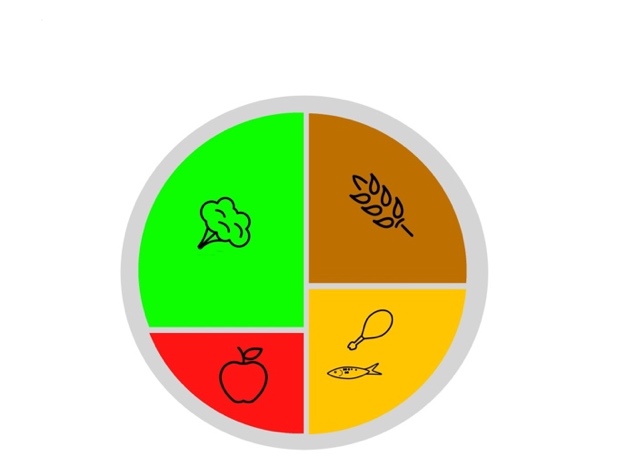 |  |  |
| **2** | **MHLH7ACT** | What is your personal reaction to the message? | 1: No action planned  2: Intention/plans to change personal behavior  3: Intention/plans to reinforce personal behavior  4: Intention/plans to take public action to change or reinforce behavior of others  5: Intention/plans to take public action and change or reinforce behavior of self and others | IF 1, Scored = 0.  IF 2 or 3, Scored = 1.  IF 4, Scored = 2.  IF 5, Scored = 3. |
|  |  | *The following is an image of a store-front in a low-income community. Use the image below to answer the next question.*  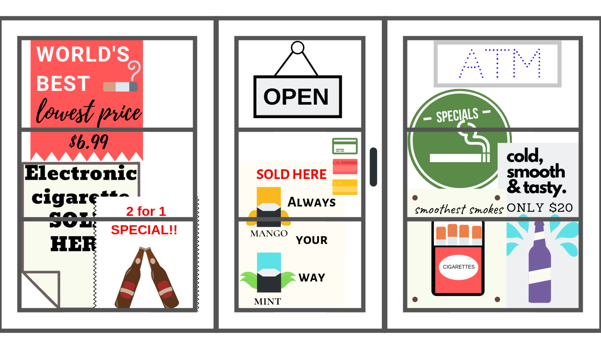 |  |  |
| **3** | **MHLH8ACT** | What is your personal reaction to the message? | 1: No action planned  2: Intention/plans to change personal behavior  3: Intention/plans to reinforce personal behavior  4: Intention/plans to take public action to change or reinforce behavior of others  5: Intention/plans to take public action and change or reinforce behavior of self and others | IF 1, Scored = 0.  IF 2 or 3, Scored = 1.  IF 4, Scored = 2.  IF 5, Scored = 3. |
