# Supplementary material for: Development and Validation of the Adolescent Media Health Literacy Scales: Rasch Measurement Model Approach
Source: JMIR Pediatr Parent. 2022 Apr 15;5(2):e35067. doi: 10.2196/35067 (PMC9055475; doi:10.2196/35067)
Supplement: Multimedia Appendix 4 [file pediatrics_v5i2e35067_app4.docx]

Appendix IV. Media Health Literacy Scales – Short Form

|  | **VARIABLE NAME** | **DESCRIPTION** | **ANSWER CHOICES** | **SCORING** |
| --- | --- | --- | --- | --- |
|  |  | *Use the image below to answer the next 5 questions.*  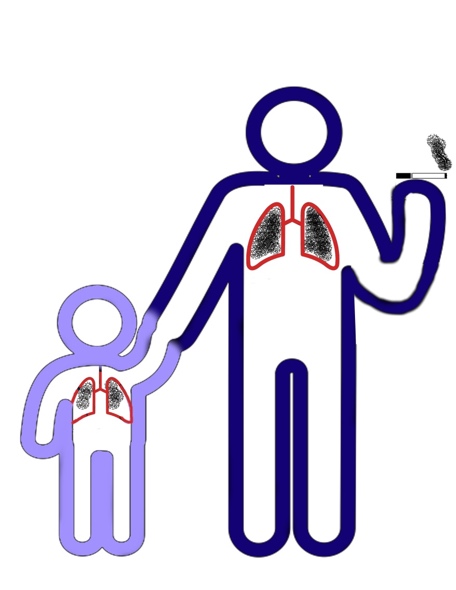 |  |  |
| **1** | **MHLH6REC** | Is there a health-related message in the picture? |  | 1: Yes  0: No |
| **2** | **MHLH6CON** | What behavior is the message about? | 1: Parent/child relationships  2: Smoking  3: Environment  4: Hand washing | IF 1, 3, or 4, Scored = 0  IF 2, Scored = 1 |
| **3** | **MHLH6INT** | What is the intent of the image? | 1: To make someone stop smoking  2: To make someone start smoking  3: To effect no change/to do nothing | IF 2 or 3, Scored = 0  IF 1, Scored =1 |
| **4** | **MHLH6INF** | To what extent do you think viewing of the message could influence individuals’ behavior? | 1: No influence  2: Some influence  3: A lot of influence | IF 1, Scored = 0  IF 2 or 3, Scored = 1 |
| **5** | **MHLH6AGR** | To what extent do you agree with the content of the message in the picture? | 1: Strongly disagree  2: Disagree  3: Neither agree or disagree  4: Agree  5: Strongly agree | IF 1, Scored = 0.  IF 2, Scored = 1.  IF 3, Scored = 2.  IF 4, Scored = 3.  IF 5, Scored = 4. |
|  |  | *Use the image below to answer the next 5 questions.*  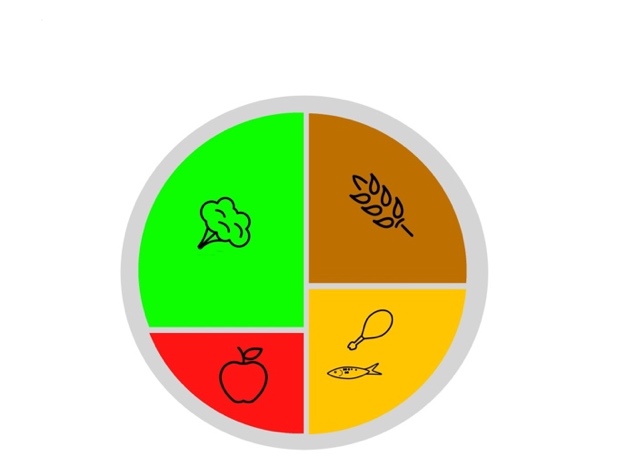 |  |  |
| **6** | **MHLH7REC** | Is there a health-related message in the picture? |  | 1: Yes  0: No |
| **7** | **MHLH7CON** | What behavior is the message about? | 1: Eating  2: Physical activity  3: Sleep  4: Alcohol use  5: Colors  6: Fractions | IF 1, Scored = 1.  IF 2, 3, 4, 5, or 6, Scored = 0. |
| **8** | **MHLH7INT** | What is the intent of the message? | 1: To show people the right portions  2: To show people which foods to eat  3: To effect no change/to do nothing | IF 1, Scored = 1.  IF 2 or 3, Scored = 0. |
| **9** | **MHLH7INF** | To what extent do you think viewing of the message could influence individuals’ behavior? | 1: No influence  2: Some influence  3: A lot of influence | IF 1, Scored = 0  IF 2 or 3, Scored = 1 |
| **10** | **MHLH7AGR** | To what extent do you agree with the content of the message in the picture? | 1: Strongly disagree  2: Disagree  3: Neither agree or disagree  4: Agree  5: Strongly agree | IF 1, Scored = 0.  IF 2, Scored = 1.  IF 3, Scored = 2.  IF 4, Scored = 3.  IF 5, Scored = 4. |
|  |  | *The following is an image of a store-front in a low-income community. Use the image below to answer the next 2 questions.*  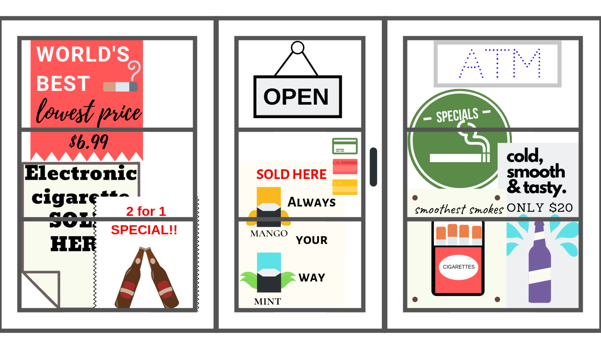 |  |  |
| **11** | **MHLH8REC** | Is there a health-related message in the picture? |  | 1: Yes  0: No |
| **12** | **MHLH8INF** | To what extent do you think viewing of the storefront window could influence individuals’ behavior? | 1: No influence  2: Some influence  3: A lot of influence | IF 1, Scored = 0  IF 2 or 3, Scored = 1 |
