# Supplementary material for: Development and Validation of the Adolescent Media Health Literacy Scales: Rasch Measurement Model Approach
Source: JMIR Pediatr Parent. 2022 Apr 15;5(2):e35067. doi: 10.2196/35067 (PMC9055475; doi:10.2196/35067)
Supplement: Multimedia Appendix 5 [file pediatrics_v5i2e35067_app5.docx]

Appendix V. Supplementary Tables

Supplemental Table 1. Standardized residual correlations of items (Q_3_) on the Recognition/Identification Scale

| Item | 1 | 2 | 3 | 4 | 5 | 6 | 7 | 8 | 9 |
| --- | --- | --- | --- | --- | --- | --- | --- | --- | --- |
| 1 MHLH1REC | 1.00 |  |  |  |  |  |  |  |  |
| 2 MHLH2REC | -.02 | 1.00 |  |  |  |  |  |  |  |
| 3 MHLH3REC | -.23 | -.07 | 1.00 |  |  |  |  |  |  |
| 4 MHLH4REC | -.11 | -.13 | <-.01 | 1.00 |  |  |  |  |  |
| 5MHLH6REC | .05 | .17 | -.18 | -.13 | 1.00 |  |  |  |  |
| 6 MHLH7REC | -.09 | .12 | -.17 | .08 | -.08 | 1.00 |  |  |  |
| 7 MHLH8REC | -.22 | -.10 | -.20 | -.17 | <.01 | -.08 | 1.00 |  |  |
| 8 MHLH9REC | -.27 | -.12 | -.06 | -.12 | -.10 | -.08 | -.18 | 1.00 |  |
| 9 MHLH10REC | -.24 | -.12 | -.10 | -.19 | -.04 | -.09 | -.30 | -.04 | 1.00 |

Supplemental Table 2. Standardized residual correlations of items (Q_3_) on the Influence/Critical Analysis Scale

| Item | 1 | 2 | 3 | 4 | 5 | 6 | 7 | 8 | 9 |
| --- | --- | --- | --- | --- | --- | --- | --- | --- | --- |
| 1 MHLH6CON | 1.00 |  |  |  |  |  |  |  |  |
| 2 MHLH6INT | -.05 | 1.00 |  |  |  |  |  |  |  |
| 3 MHLH7CON | <.01 | .04 | 1.00 |  |  |  |  |  |  |
| 4 MHLH7INT | -.01 | .04 | .13 | 1.00 |  |  |  |  |  |
| 5 MHLH6INF | -.03 | .09 | -.11 | -.08 | 1.00 |  |  |  |  |
| 6 MHLH7INF | -.08 | <-.01 | -.06 | -.14 | .05 | 1.00 |  |  |  |
| 7 MHLH8INF | -.08 | .02 | .10 | -.01 | .15 | -.05 | 1.00 |  |  |
| 8 MHLH6AGR | -.21 | -.03 | -.20 | -.26 | -.07 | -.19 | -.19 | 1.00 |  |
| 9 MHLH7AGR | -.18 | -.29 | -.22 | -.26 | -.19 | -.03 | -.18 | -.49 | 1.00 |

Supplemental Table 3. Standardized residual correlations of items (Q_3_) on the Action/Reaction Scale

| Item | 1 | 2 | 3 |
| --- | --- | --- | --- |
| 1 MHLH6ACT | 1.00 |  |  |
| 2 MHLH7ACT | -.42 | 1.00 |  |
| 3 MHLH8ACT | -.60 | -.47 | 1.00 |

Supplemental Table 4. Standardized residual correlations of items (Q_3_) on the Media Health Literacy Scales – Short Form

| Item | 1 | 2 | 3 | 4 | 5 | 6 | 7 | 8 | 9 | 10 | 11 | 12 |
| --- | --- | --- | --- | --- | --- | --- | --- | --- | --- | --- | --- | --- |
| 1 MHLH6CON | 1.00 |  |  |  |  |  |  |  |  |  |  |  |
| 2 MHLH6INT | -.04 | 1.00 |  |  |  |  |  |  |  |  |  |  |
| 3 MHLH7CON | .01 | .04 | 1.00 |  |  |  |  |  |  |  |  |  |
| 4 MHLH7INT | -.01 | .04 | .10 | 1.00 |  |  |  |  |  |  |  |  |
| 5 MHLH6INF | -.02 | .11 | -.11 | -.08 | 1.00 |  |  |  |  |  |  |  |
| 6 MHLH7INF | -.09 | -.02 | -.07 | -.15 | .05 | 1.00 |  |  |  |  |  |  |
| 7 MHLH8INF | -.09 | .04 | .12 | -.02 | .14 | -.05 | 1.00 |  |  |  |  |  |
| 8 MHLH6AGR | -.17 | -.03 | -.20 | -.21 | -.07 | -.15 | -.16 | 1.00 |  |  |  |  |
| 9 MHLH7AGR | -.16 | -.23 | -.20 | -.25 | -.14 | -.05 | -.18 | -.29 | 1.00 |  |  |  |
| 10 MHLH6REC | .02 | .12 | .08 | .02 | .01 | -.01 | -.02 | -.21 | -.19 | 1.00 |  |  |
| 11 MHLH7REC | -.02 | -.03 | .03 | .01 | -.11 | .004 | -.03 | -.18 | -.03 | .04 | 1.00 |  |
| 12 MHLH8REC | -.04 | -.12 | .004 | -.03 | -.02 | .03 | .01 | -.33 | -.25 | .09 | -.01 | 1.00 |

Supplemental Table 5. Reliability statistics of all Media Health Literacy Scales.

|  | Item-separation reliability | Wright sample independent reliability | Kuder-Richardson Formula 20 |
| --- | --- | --- | --- |
| Recognition/Identification | .98 | .80 | .74 |
| Influence/Critical Analysis | .96 | .90 | .91 |
| Action/Reaction | .82 | .80 | .71 |
| Media Health Literacy Scale- Short Form | .96 | .90 | .93 |
